# Utility of vector flow mapping technology in quantitative assessment of carotid wall shear stress in hypertensive patients: A preliminary study

**DOI:** 10.3389/fcvm.2022.967763

**Published:** 2022-10-28

**Authors:** Lan He, Yundan Cai, Yuhong Feng, Wenwen Wang, Tienan Feng, E. Shen, Shaoling Yang

**Affiliations:** ^1^Department of Ultrasound Medicine, Shanghai Eighth People’s Hospital, Shanghai, China; ^2^Department of Ultrasound Medicine, Shanghai Sixth People’s Hospital, Shanghai, China; ^3^FUJIFILM Healthcare (Guangzhou), Co., Ltd., Guangzhou, China; ^4^Clinical Research Institute, Shanghai Jiao Tong University School of Medicine, Shanghai, China; ^5^Department of Ultrasound Medicine, Chest Hospital Affiliated to Shanghai Jiao Tong University, Shanghai, China

**Keywords:** vascular VFM, wall shear stress, carotid artery, hypertension, carotid atherosclerosis

## Abstract

**Background:**

Blood flowing in the arterial lumen acts on the surface of the vessel wall to form wall shear stress (WSS). To date, there has been limited research on the utility of non-invasive technology in the accurate quantification of carotid WSS in patients with hypertension (HP).

**Objective:**

The present study aimed to explore the usage of vascular vector flow mapping (VFM) in the quantitative assessment of carotid WSS in hypertensive patients at an early stage and to validate its clinical utility.

**Methods:**

A total of 50 individuals confirmed without carotid plaques were grouped into a HP group (*n* = 25) and a control (CON) group (*n* = 25) according to blood pressure. An ALOKA LISENDO 880 Color Doppler Ultrasound with a L441 3–15 MHZ probe was used to obtain a longitudinal section scan to determine the regions of interests (ROIs) of the common carotid artery. VFM-based WSS measurements were obtained by selecting the ROI with optimal image quality from three full cardiac cycles. WSS-derived measurements, including WSS_max_, WSS_min_, and WSS_mean_, were analyzed and compared between the HP and CON groups. In addition, the correlations between WSS-derived measurements and the carotid artery intima-media thickness (IMT) were also analyzed.

**Results:**

There were significant statistical differences in WSS_max_ and WSS_mean_ between patients in the HP and CON groups. Specifically, the HP group had significantly decreased WSS_max_ and WSS_mean_ compared to the CON group (WSS_max_: 1.781 ± 0.305 Pa vs. 2.286 ± 0.257 Pa; WSS_mean_: 1.276 ± 0.333 Pa vs. 1.599 ± 0.293 Pa, both *p* < 0.001). However, there was no statistical difference in WSS_min_ between the groups (0.79 ± 0.36 vs. 0.99 ± 0.42, *p* = 0.080). Additionally, Spearman’s correlation analysis indicated that the WSS-derived parameters were negatively correlated with the IMT (*p* < 0.001).

**Conclusion:**

Vascular VFM technology shows promising results in the quantitative assessment of difference in hemodynamics of the vascular flow field between patients with HP and normal controls. Difference in WSS may serve as a potential predictor for the development of arteriosclerosis risks.

## Introduction

Hypertension (HP) is a serious health condition that is common among adults with 200 million patients reported in China with an annual increment rate of 3–4 million (1). HP promotes the progression of carotid atherosclerosis, which acts as an independent risk factor for various cardiovascular and cerebrovascular diseases (2, 3), including ischemic stroke (4). At present, ultrasound, computed tomography, and magnetic resonance imaging (MRI) are used to diagnose carotid artery structural lesions, and they have been used in comprehensive studies to evaluate carotid artery-related characteristics, such as intima-media thickening (IMT) (5), atherosclerotic plaque formation (6), plaque-induced lumen stenosis, and plaque stability (7). Early diagnosis of carotid artery damage has become an active research topic due to the advances in imaging technology, such as pulse wave technology, vascular echoelasticity technology, and shear wave viscoelasticity imaging technology (8–11). These approaches provide quantitative measurements of carotid artery injury from structural lesions to function assessment. However, it is worth mentioning that these methods often lack the evaluation of vascular hemodynamics, which limit their use in predicting development and progression of early vascular lesions.

Hemodynamics play an important role in the development and progression of blood vessels because they regulate vascular structure and modulate angiogenesis, thereby affecting atherosclerosis, aneurysms, narrow expansion, and arterial deformity (12). Blood flow-induced shear stress, an indicator of vascular hemodynamics, is currently used in atherosclerosis risk assessment. In particular, wall shear stress (WSS), a quantitative parameter of fluid mechanics, is the frictional force acting on a unit area of the blood vessel wall surface during blood flow with a physical unit of Pa (13, 14). WSS can be measured using vector flow mapping (VFM) technology, which is a non-invasive imaging approach to analyze blood flow-induced hemodynamics that has been increasingly used in cardiovascular imaging (15, 16). Briefly, VFM combines color Doppler imaging and speckle-tracking analysis, providing quantitative and subjective measurements, including the blood flow velocity vector and WSS. Although low WSS is closely associated with early atherosclerotic lesions, there is limited research on the application of vascular VFM technology in the quantitative assessment of WSS before carotid atherosclerotic plaque formation in hypertensive patients.

Previous studies have used intravascular ultrasound (17), computed tomography angiography (18), or MRI (19) to assess the effect of shear stress on coronary plaque progression and changes in plaque composition. Intravascular ultrasound is regarded as an invasive operation, and images acquired by computed tomography angiography and MRI need to be reconstructed before post-analysis. Additionally, attempts have been made to incorporate four-dimensional blood-magnetic resonance imaging (4D Flow MRI) into the analysis of carotid blood flow dynamics (20, 21). However, these results are often unsatisfactory due to the limited spatial resolution and time-consuming scanning procedure. Thus, there is high demand to utilize non-invasive and user-friendly technology in early assessment of vascular function. Therefore, the purpose of the present study was to evaluate VFM-based WSS measurements for the quantitative assessment of vascular function and to determine their role in early prediction of carotid artery lesions caused by HP. This preliminary study aimed to provide a novel approach for early clinical evaluation and may guide the selection and implementation of early prevention and clinical treatment methods.

## Materials and methods

### Ethics statement

This study was approved by the Ethics Committee of Shanghai Eighth People’s Hospital. Informed consent was waived due to the retrospective nature of the study.

### Patient enrollment

We retrospectively collected 50 individuals who visited the Ultrasound Department in our hospital between March 2021 and May 2021. According to the Chinese Guidelines for the Prevention and Treatment of HP (Revised in 2018) (22), HP was diagnosed when systolic blood pressure ≥ 140 mmHg and/or diastolic blood pressure ≥ 90 mmHg. Thus, all patients were divided into the following two groups: HP group (*n* = 25) and control (CON) group (*n* = 25). Patients who met the following criteria were excluded from the study: (1) Carotid atherosclerotic plaque formation; (2) diagnosis of cardiovascular and cerebrovascular diseases; (3) abnormal liver and kidney function; and (4) metabolic abnormalities, such as hyperlipidemia, hyperglycemia, and hyperuricemia.

### Scanning protocol

**FIGURE 1:**
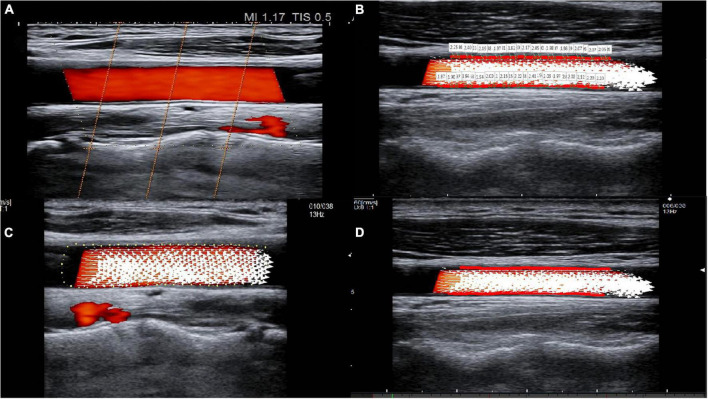
The visualization of common carotid artery blood flow and the quantitative calculation of WSS. **(A)** Three sound beams intersecting with the color Doppler appear when the VFM button is activated. Cross-beam vascular VFM creates a starting point to replace the vessel wall, and it obtains blood flow velocity information perpendicular to the direction of the color Doppler. **(B)** Tracing of the intima of the anterior and posterior walls of the common carotid artery and visualization of the WSS at each point of the anterior and posterior walls of the carotid artery. **(C)** The white arrow represents the blood flow velocity vector of the carotid artery. **(D)** The red line(s) on the vessel wall indicates that the WSS is high.

The ALOKA LISENDO 880 color Doppler ultrasound diagnostic apparatus (FUJIFILM Healthcare (Guangzhou) Co., Ltd., Japan) was used in this study. The L441 3–15 MHz probe was used to scan the common carotid artery in a longitudinal orientation. Patients were firstly connected to the electrocardiogram during the examination. When using the conventional two-dimensional ultrasound, the long axis of the common carotid artery was adjusted to keep the long axis parallel to the skin. The color Doppler adjusted the color sampling frame angle between the probe and the carotid artery between 0° and 30°. The captured image frame rate was ≥ 10 frames, and the color Doppler flow mapping dynamic range was set at the lowest possible magnitude within the limit of the first-order aliasing correction. By enabling the VFM vascular key on the color Doppler after routine measurement, three sound beams intersecting with the color Doppler appeared, and the position of the sampling frame was fixed in the ROIs in the middle segment of the common carotid artery (Figure 1A). We collected dynamic images of three full cardiac cycles by tracing the intima of the anterior and posterior walls of the common carotid artery. The WSS at each point of the anterior and posterior walls of the carotid artery was visualized (Figure 1B). The white arrow represents the blood flow velocity vector of the carotid artery (Figure 1C).

### Vector flow mapping -based wall shear stress parameters and intima-media thickness measurements

WSS was calculated in each frame and at each measured point. The cardiac cycle with the best image quality among the three cardiac cycles was selected for analysis. In each cardiac cycle, there were approximately 10–15 frames, and in each frame, there were approximately 38–50 points. The WSS paraments, including WSS_max_, WSS_min_, and WSS_mean_, were obtained after averaging the parameters across all frames and all points in a cardiac cycle. The color scale on the carotid artery wall intuitively reflects the high and low levels of WSS parameters, while red line(s) on the vessel wall indicate a high WSS (Figure 1D).

WSS was automatically calculated using the following analytical formula embedded in the software:


$$W\,a\,l\,l\,S\,h\,e\,a\,r\,S\,t\,r\,e\,s\,s\,\left(W\,S\,S\right)=\mu \,\left(\frac{d\,v}{d\,y}\right)$$

$$\mu =4.0\times 10^{-3}\,\left(N\cdot s/m^{2}\right)$$

d⁢v/d⁢y:w⁢a⁢l⁢l⁢s⁢h⁢e⁢a⁢r⁢r⁢a⁢t⁢e

These WSS parameters were analyzed at the patient level for later comparison. In addition, the carotid IMT measurements were calculated using the vertical distance from the superior intima to the superior adventitia at partially enlarged longitudinal sections that were 1.0–1.5 cm below the level of the carotid bifurcation. The final values were obtained by taking the average of three independent measurements where the common carotid artery IMT ≤ 1.0 mm indicates normal, IMT ≥ 1.1 mm indicates IMT thickening, and IMT ≥ 1.5 mm indicates plaque formation (23).

### Statistical analysis

Statistical analysis was performed using SPSS 23.0 software (IBM Corp, Armonk, NY, USA), GraphPad Prism 8.4.3 (San Diego, CA). Descriptive statistics of patient characteristics were presented as frequencies and percentages for categorical variables, and they were presented as means and standard deviation (SD) or as median with interquartile range (IQR) for continuous variables. The normality of the data distribution was assessed by the Shapiro–Wilk Test. Differences in categorical variables between the HP and CON groups were analyzed using the Chi-squared test or Fisher’s exact test as appropriate. Differences in continuous variables between groups were analyzed using the independent *t*-test or Mann–Whitney *U*-test. Correlations between the WSS and the IMT quantifications were analyzed with the Spearman’s correlation coefficient (r). A *p*-value less than 0.05 was considered statistically significant.

## Results

### Patient characteristics

In total, 50 patients were included in the analysis with 25 patients in the HP group and 25 patients in the CON group. As shown in Table 1, there was no statistical difference in sex, age, and heart rate between the two groups. However, there were significant statistical differences in body surface area, systolic blood pressure, diastolic blood pressure, and IMT between the HP and CON groups (all *p* < 0.05).

**TABLE 1:** General parameters of the HP and CON groups.

General parameters		HP group	CON group	*P*
Cases		25	25	
Sex	Male	13	15	0.395
	Female	12	10	
Age (years)		58.32 ± 17.03	51.68 ± 19.02	0.200
Heart rate (bpm)		69.24 ± 5.88	69.64 ± 7.32	0.832
BSA (kg/m^2^)		1.83 ± 0.17	1.69 ± 0.14	0.001*
Systolic blood pressure (mmHg)		168.40 ± 10.18	118.56 ± 7.25	< 0.001*
Diastolic blood pressure (mmHg)		94.00 ± 5.40	79.52 ± 3.62	< 0.001*
IMT (mm)		1.10 ± 0.11	0.86 ± 0.14	< 0.001*

BSA, body surface area; IMT, intima-media thickness. *Indicates statistically significant difference assessed by independent *t*-test or Mann–Whitney *U*-test.

### Wall shear stress parameters of the hypertension and control groups

The VFM-based WSS paraments, including WSS_max_, WSS_min_ and WSS_mean_, were calculated and compared between the two groups. Compared to the CON group, patients in the HP group had significantly decreased values of WSS_max_ and WSS_mean_ (both *p* < 0.05), but there was no significant difference in WSS_min_ between the two groups (Table 2).

**TABLE 2:** Carotid WSS parameters of the HP and CON groups.

WSS (Pa)	HP group	CON group	*P*
Case	25	25	
Max	1.78 ± 0.31	2.29 ± 0.26	<0.001*
Min	0.79 ± 0.36	0.99 ± 0.42	0.080
Mean	1.28 ± 0.33	1.60 ± 0.29	0.001*

HP, hypertension; WSS, wall shear stress. *Indicates statistically significant difference assessed by independent *t*-test or Mann–Whitney *U*-test.

### Correlation between wall shear stress parameters and intima-media thickening

**FIGURE 2:**
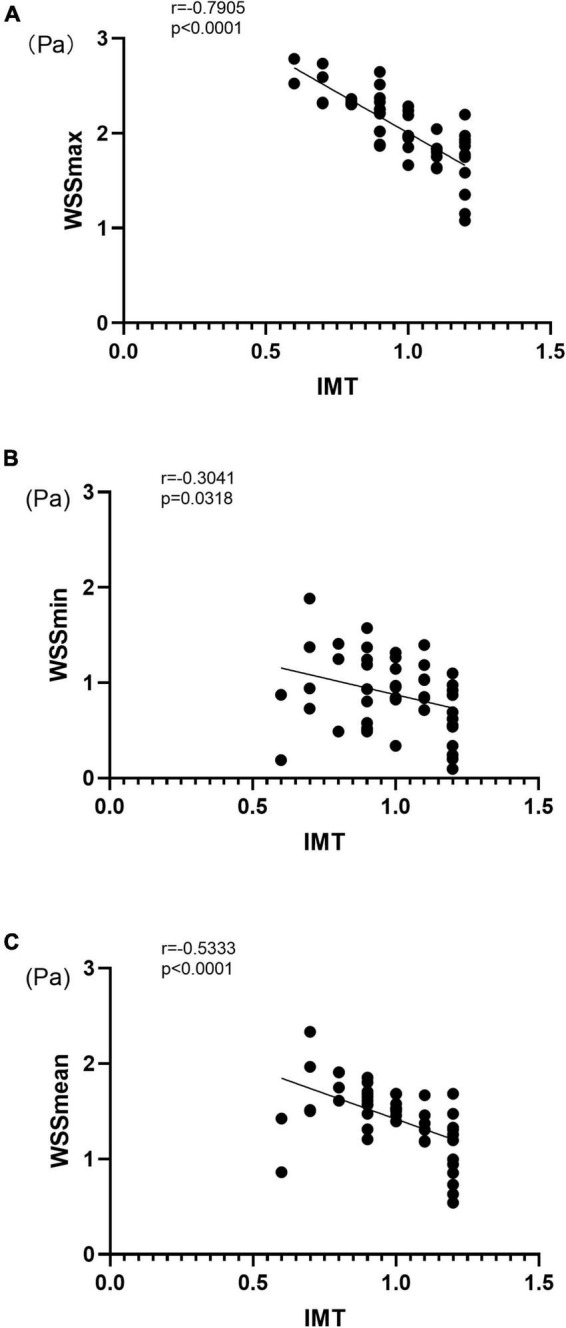
Correlation between carotid artery VFM-based WSS parameters and IMT for WSS_max_ **(A)**, WSS_min_ **(B)**, and WSS_mean_ **(C)**. IMT, intima-media thickness; WSS, wall shear stress; r, Spearman’s correlation coefficient.

To evaluate the relationship between the WSS parameters and IMT, we applied correlation analysis using Spearman’s coefficient (r). As shown in Figure 2, WSS_max_, WSS_min_, and WSS_mean_ were negatively correlated with IMT with Spearman’s correlation coefficients of –0.790, –0.304, and –0.533, respectively (all *p* < 0.05) (Figure 2). We also evaluated the correlation between WSS and IMT between the HP and CON groups and found that WSS_max_ and WSS_mean_ were negatively correlated with IMT in the HP group (both *p* < 0.05) (Supplementary Figure 1).

## Discussion

In the present study, advanced vascular VFM technology was used to quantify and visualize the hemodynamic differences of carotid artery flow field in hypertensive and non-hypertensive patients. WSS-derived parameters including, WSS_max_, WSS_mean_, and WSS_min_, were closely associated with carotid vascular deformation in hypertensive patients. These findings showed that the WSS_max_ and WSS_mean_ were significantly decreased in the HP group compared to the CON group, while WSS_min_ had no significant difference between the groups. Additionally, negative correlations were observed between carotid artery WSS parameters and IMT.

This preliminary study is the first reported attempt to evaluate the quantitative WSS parameters generated by vascular VFM technology and their differences between patients with and without HP. The results demonstrated differences in the hemodynamics of the carotid artery lumen flow field in patients with HP. It has been previously demonstrated that HP status induces complex hemodynamic conditions of the blood flow in the carotid artery (24, 25). Particularly, the blood flow impacts the carotid artery wall, forming shear stress that affects the vascular wall (26), which may result in vascular endothelial cell damage and platelet aggregation at the injury site. Zhang et al. (20) used a 4D flow MRI approach and demonstrated that low shear stress is significantly correlated with increased atherosclerosis progression rate. Additionally, blood pressure fluctuates in some patients with HP, which may increase blood flow resistance and promote carotid artery remodeling (27, 28). The impact force of the blood vessel wall aggravates the damage of carotid endothelial cells, promotes the thickening of the carotid intima-media, and promotes the formation of carotid atherosclerotic plaques (29, 30). The formation of carotid atherosclerotic plaques affects the compliance of carotid arteries, which in turn aggravates the degree of HP (31). This interaction further contributes to increased risks of cardiovascular and cerebrovascular diseases (32). The present results were consistent well with a previous study by Wei et al. (33). The researchers used speckle-tracking strain imaging to demonstrate that the local shear stress is associated with carotid vascular deformation in patients with HP before the occurrence of any clinical symptoms or atherosclerotic plaques. However, the measurements of WSS in their study were based on assumed parabolic velocity profile, which may have led to an underestimation of WSS.

**FIGURE 3:**
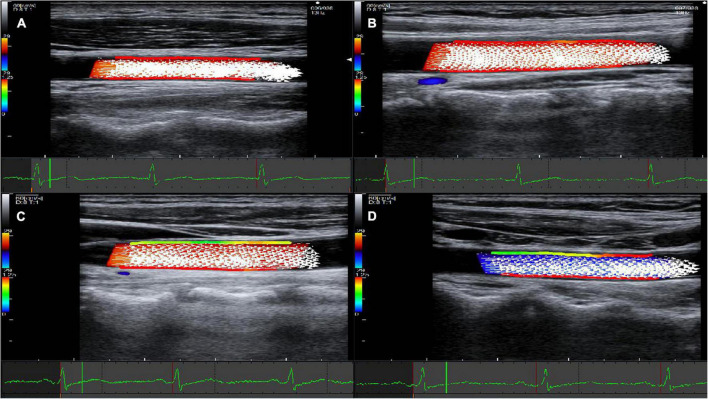
Representative images acquired using vascular VFM technology. **(A)** Fifty-five year-old male in the CON group. **(B)** Sixty-three year-old female in the CON group. **(C)** Fifty-five year-old female in the HP group. **(D)** Fifty nine -Year-old male in the HP group. Frames that correspond to the systolic period of the cardiac cycle were selected for all four patients. The red lines on the blood vessel wall indicate high wall shear stress, while the green lines indicate low wall shear stress. The front and rear walls of the carotid artery show high WSS in both **(A,B)**, and the WSS on the anterior walls of the carotid artery was significantly lower in both **(C,D)**.

The ideal motion state of blood flow is concentric and stratified, but realistic pattern of hemodynamics can be complicated. For example, the fluctuation of the shear stress on the vessel wall, the change of the blood flow from stable laminar flow to non-laminar flow state, turbulent flow, and boundary hemodynamic factors are closely related to the occurrence of arteriosclerosis (34, 35). Thus, it is of great clinical significance to accurately and objectively assess the blood flow status. The technical innovation of vascular VFM technology lies in the color Doppler method, which was used to measure the vascular hemodynamics in the present study. Specifically, the system uses color Doppler and the original three pulsed Doppler cross-beams combined with two-dimensional speckle-tracking technology. By sending and receiving cross-beams, vascular VFM creates a starting point that substitutes for the vessel wall and acquires blood velocity information perpendicular to the direction of color Doppler. The WSS size is calculated frame-by-frame and point-by-point, resulting in good temporal and spatial resolution, allowing accurate analysis of the WSS when considering the complexity pattern of blood flows in the carotid artery lumen. Vascular VFM technology visually displayed the magnitude of WSS in the HP and CON groups with red lines on the blood vessel wall indicating high WSS in the CON group and green lines on the blood vessel wall indicating low WSS in the HP group (Figure 3).

The present findings revealed a negative correlation between the WSS parameters and carotid artery IMT, which may suggest that differences in WSS are closely associated with changes and remodeling of the vascular intima, and these results may also suggest that the intima are sensitive to the local biomechanical environment, especially to the differences in WSS. Similarly, Morbiducci et al. (36) used magnetic resonance angiography and reported a significant association between low WSS and maximal IMT at a 60-month follow-up after carotid endarterectomy. Yang et al. (37) used common carotid artery ultrasound and also demonstrated a strong correlation between low carotid artery WSS and increased IMT thickness. These findings suggest that low WSS is associated with IMT, which may further stimulate changes in carotid hemodynamics in patients with HP.

The present study had several limitations. First, the WSS parameters were measured on patients before formation of carotid plaques. A longitudinal study on patients with HP-induced carotid atherosclerosis should be performed to evaluate VFM-based WSS parameters in predicting the formation of carotid plaques. Second, there was a lack of quantitative information for the blood flow velocity vector, blood flow velocity gradient, and lumen diameter, which are related to the thickness of WSS and IMT to a certain extent. Finally, the present study was a single-center study with limited sample size. However, the power analysis was performed using G Power software by using the correlation coefficient between WSS and IMT with a significance level of 0.05, and the power was greater than 0.8, indicating sufficient power for the analysis. Future work will be performed using a larger sample size and comprehensive subgroup analysis.

## Conclusion

The vascular VFM technique shows promising results in the quantitative assessment of different aspects of vascular flow field hemodynamics in hypertensive and non-hypertensive patients. WSS parameters in hypertensive patients may be used in clinical evaluation of vascular function and may serve as a potential predictor for the development of the arteriosclerosis risks.

## Data availability statement

The original data presented in the study can be accessed upon reasonable request.

## Ethics statement

The studies involving human participants were reviewed and approved by the Ethics Committee of Shanghai Eighth People’s Hospital. Written informed consent for participation was not required for this study in accordance with the national legislation and the institutional requirements. Written informed consent was not obtained from the individual(s) for the publication of any potentially identifiable images or data included in this article.

## Author contributions

LH collected the data, analyzed the data, and drafted the manuscript. YF conceived the idea of the review. YC and WW collected the data. TF analyzed the data. SY and ES critically revised the manuscript. All authors read and approved the final manuscript.
