# Supplementary material for: Utility of vector flow mapping technology in quantitative assessment of carotid wall shear stress in hypertensive patients: A preliminary study
Source: Front Cardiovasc Med. 2022 Oct 28;9:967763. doi: 10.3389/fcvm.2022.967763 (PMC9649775; doi:10.3389/fcvm.2022.967763)
Supplement: Supplementary file 1 [file Presentation_1.zip › supplementary material Presentation1.0.PDF]

Supplementary Material

Supplementary Data

Correlation between WSS parameters and IMT in the HP and CON groups

As shown in Supplementary Figure S1, the carotid artery WSS_{max} and WSS_{mean} were negatively correlated with IMT in the HP group with Spearman's correlation coefficients of -0.480 and -0.587, respectively ($p < 0.05$). For the CON group, however, only carotid artery WSS_{max} was negatively correlated with IMT with a Spearman's correlation coefficient of -0.761 ($p < 0.05$). No strong correlation was found for the WSS_{mean} and WSS_{min} parameters with IMT as indicated by the Spearman's correlation coefficients of -0.109 and -0.091, respectively ($p > 0.05$) .

Supplementary Figures

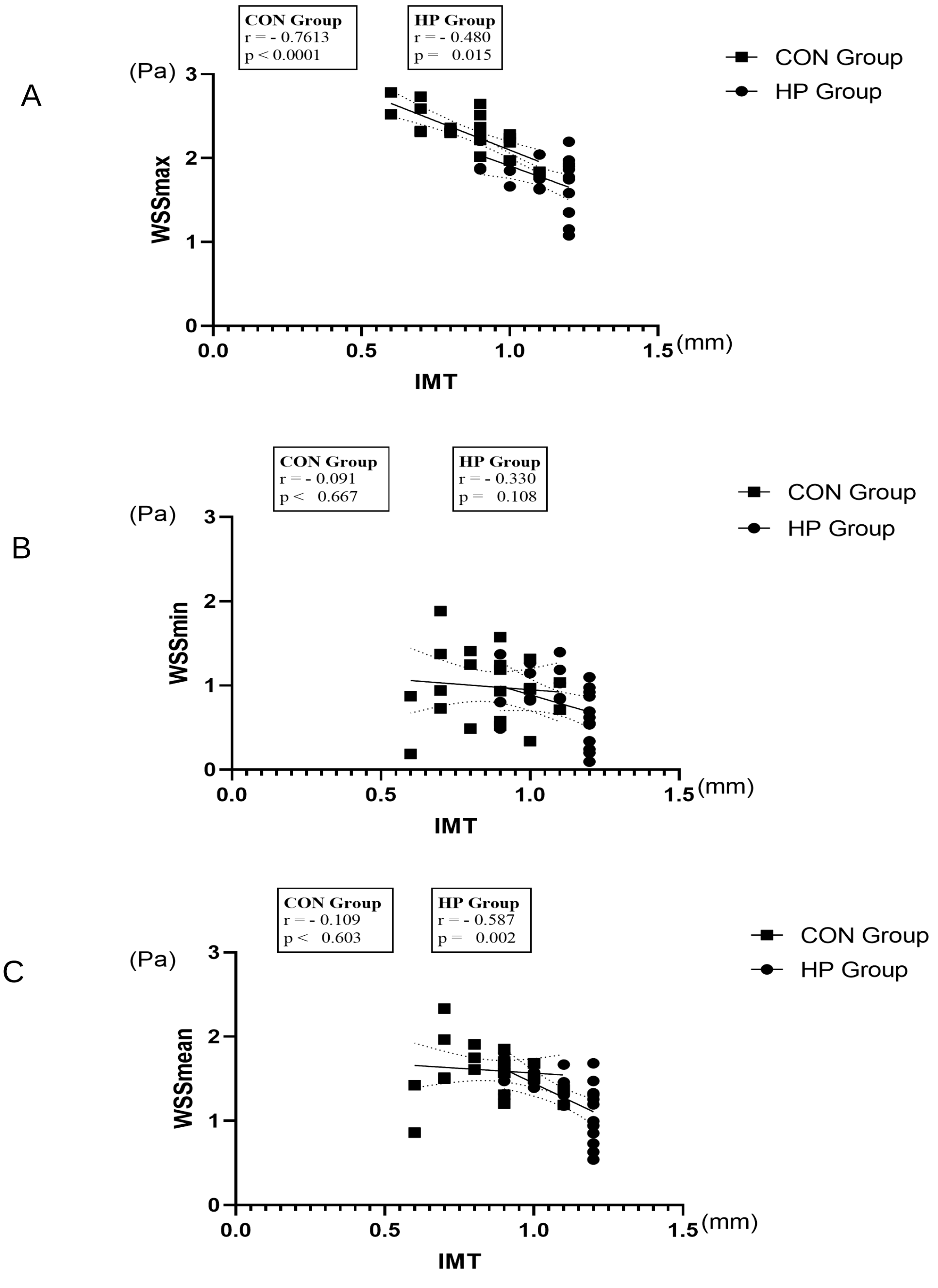

Supplementary Figure S1. Correlation of IMT with carotid artery WSS$_{max}$ (A), WSS$_{min}$ (B), and WSS$_{mean}$ (C) in the HP and CON groups. Abbreviations: IMT, intima-media thickness; WSS, wall shear stress.
